# Scoring systems for outcome prediction in patients with perforated peptic ulcer

**DOI:** 10.1186/1757-7241-21-25

**Published:** 2013-04-10

**Authors:** Kenneth Thorsen, Jon Arne Søreide, Kjetil Søreide

**Affiliations:** 1 Department of Gastrointestinal Surgery, Stavanger University Hospital, POB 8100, Stavanger, N, 4068, Norway; 2 Department of Clinical Medicine, University of Bergen, Bergen, Norway

**Keywords:** Perforated peptic ulcer, Scoring systems, Outcome prediction, Mortality, Morbidity

## Abstract

**Background:**

Patients with perforated peptic ulcer (PPU) often present with acute, severe illness that carries a high risk for morbidity and mortality. Mortality ranges from 3-40% and several prognostic scoring systems have been suggested. The aim of this study was to review the available scoring systems for PPU patients, and to assert if there is evidence to prefer one to the other.

**Material and methods:**

We searched PubMed for the mesh terms “perforated peptic ulcer”, “scoring systems”, “risk factors”, ”outcome prediction”, “mortality”, ”morbidity” and the combinations of these terms. In addition to relevant scores introduced in the past (e.g. Boey score), we included recent studies published between January 2000 and December 2012) that reported on scoring systems for prediction of morbidity and mortality in PPU patients.

**Results:**

A total of ten different scoring systems used to predict outcome in PPU patients were identified; the Boey score, the Hacettepe score, the Jabalpur score the peptic ulcer perforation (PULP) score, the ASA score, the Charlson comorbidity index, the sepsis score, the Mannheim Peritonitis Index (MPI), the Acute physiology and chronic health evaluation II (APACHE II), the simplified acute physiology score II (SAPS II), the Mortality probability models II (MPM II), the Physiological and Operative Severity Score for the enumeration of Mortality and Morbidity physical sub-score (POSSUM-phys score). Only four of the scores were specifically constructed for PPU patients. In five studies the accuracy of outcome prediction of different scoring systems was evaluated by receiver operating characteristics curve (ROC) analysis, and the corresponding area under the curve (AUC) among studies compared. Considerable variation in performance both between different scores and between different studies was found, with the lowest and highest AUC reported between 0.63 and 0.98, respectively.

**Conclusion:**

While the Boey score and the ASA score are most commonly used to predict outcome for PPU patients, considerable variations in accuracy for outcome prediction were shown. Other scoring systems are hampered by a lack of validation or by their complexity that precludes routine clinical use. While the PULP score seems promising it needs external validation before widespread use.

## Introduction

Peptic ulcer disease is associated with potentially life- threatening complications, including bleeding, perforation, penetration and obstruction. Perforation is the second most frequent complication after bleeding [1]. While the clinical picture of patients with perforated peptic ulcer (PPU) sometimes can be blurred by vague symptoms, most PPU patients present with overt symptoms and signs of peritonitis and eventually sepsis. Variations in the clinical presentation as well as delay in diagnosis and work-up at admission to the hospital, may potentially cause a worsening of symptoms and a deterioration of the clinical condition, with a detrimental outcome. Still, a high risk for morbidity (20-50%) and mortality (3-40%) is encountered in surgically treated PPU patients [2-7]. About every fifth patient with PPU present with signs of sepsis and by a careful preoperative assessment of the patients´ severity grade, appropriate management can be offered to achieve an optimal outcome of disease [8,9]. Currently, the ASA score and the Boey score are the most frequently used prognostic scoring systems in patients with PPU [10-14]. Yet, the ASA score is a general surgical risk score not intended for PPU patients in particular. Moreover, the external validation of the Boey score is uncertain.

The aim of this study was to review the available scoring systems used for outcome prediction in PPU patients, and to evaluate if any scoring system has advantages and predictive power to be preferred in clinical practice on this group of patients.

## Material and methods

We searched the Pub Med database by using the mesh terms; “perforated peptic ulcer”, “scoring systems for morbidity and mortality”, “risk factors”, ”outcome prediction” and the combinations of these terms. In addition we did a manual search from identified articles of relevance. We evaluated studies on PPU patients and related outcomes (morbidity and mortality), published in the English language between 2000 and 2012, and recognized the scores or combinations of scores that have been used in these studies. A few seminal articles published before 2000 were also taken into consideration. The study data were compared by a descriptive approach.

### Definitions

Receiver operating characteristics curve (ROC) is a statistical method that measures diagnostic accuracy of a test and offers graphical display of the true positives versus the false positives. Area under the curve (AUC) is used to measure the “size” of the curve of prediction composed by the graphic display between the ‘sensitivity’ and the ‘1–specificity’ relationship. AUC can range from 0.5 to 1.0 and a result of 1.0 indicates a perfect discriminatory ability [15]. An AUC value > 0.8 is considered good, a range between 0.60-0.80 is considered as moderate, and an AUC value < 0.60 is regarded as poor [16]. The ROC curve is calculated by using all possible score values as a potential cut-off value for a given outcome prediction (such as mortality), and displays the optimal cut-off point when sensitivity and specificity reaches an optimum for both values, by which the point on the ROC curved line is closest to the upper left corner on the curve.

## Results

Ten different scoring systems (Table 1) used for prediction of outcomes for PPU patients were identified, including the Boey score [14], the Hacettepe score [17] the Jabalpur score [18], the Peptic Ulcer Perforation (PULP) score [19], the American Society of Anesthesiologists (ASA) score [20], the Charlson comorbidity index [21], the sepsis score [22], the Mannheim Peritonitis Index (MPI) [23], the Acute Physiology and Chronic Health Evaluation II (APACHE II) [24], the Simplified Acute Physiology Score II (SAPS II) [25], the Mortality Probability Models II (MPM II) [26] and the Physiological and Operative Severity Score for the enumeration of Mortality and Morbidity physical sub-score (POSSUM-phys score) [27]. In five studies, comparison of various scoring systems for outcome prediction was done by ROC-analyses with reporting on the area under the curve (AUC) [10,16,18,19,22]. A few studies presented specificity and sensitivity, relative risks (RR) and odds ratios (OR), while most studies reported on performance by calculation of the chi square test.

**Table 1:** Scoring systems used for outcome prediction in perforated peptic ulcer

**Scoring systems (reference)**	**Year of report**	**Target population**	**Outcome measured**	**Parameters evaluated**
**Boey**[14]	1987	Patients with PPU	30 day mortality	Presentation ≥ or <24 hours; presence of preoperative shock; level of comorbidity.
**Hacettepe score**[17]	1992	Patients with PPU	30 day mortality	Presence of serious medical illness, acute renal failure, white blood cell count, male gender
**Jabalpur score**[18]	2003	Patients with PPU	30 day mortality	Time from perforation to operation, mean systolic blood pressure preoperatively, heart rate, serum creatinine, age, comorbidity
**PULP**[19]	2012	Patients with PPU	30 day mortality	Presentation ≥ or <24 hours; presence of preoperative shock; ASA score, presence of aids, active malignancy, liver failure; serum creatinine > 130 mmol/l
**ASA**[20]	1941	General surgical populations	Preoperative risk assessment for surgical patients	Degree of comorbidity and present systemic disease
**Charlson comorbidity index**[21]	1987	General surgical populations	Prediction of 1 year mortality for hospitalized patients	Weighting of different comorbidities
**Mannheim peritonitis index**[23]	2002	General peritonitis	Peroperative prediction of outcome in patients with peritonitis	Age, gender, organ failure, duration of peritonitis, site of perforation, diffuse peritonitis, level of exudate
**APACHE II**[24]	1985	Critically ill patients	Prediction of outcome for ICU patients	Aids, metastatic cancer, liver failure, immunosuppression, chronic renal insufficiency, haemotologic malignancy, lymphoma, leukemia, age, heart rate, systolic blood pressure, respiratory rate, temperature, GCS, WBC, creatinine, blood gas, potassium, sodium, patient origin
**SAPS II**[25]	1993	Critically ill patients	Prediction of outcome for ICU patients	Aids, metastatic cancer, haemotologic malignancy, age, heart rate, systolic blood pressure, temperature, GCS, urine output, WBC, bilirubin, urea, Potassium, sodium, Patient origin
**MPM II**[26]	1993	Critically ill patients	Prediction of outcome for ICU patients	Metastatic cancer, liver failure, chronic renal insufficiency, leukemia, age, acute renal failure, arrythmias, heart rate, GI bleeding, GCS, intracranial mass effect, cerebrovascular accident, cpr prior to admission, mechanical ventilation
**POSSUM**[37]	1991	Surgical patients	Prediction of outcome (mortality) for surgical patients	Respiratory history; cardiac signs; age; heart rate; systolic blood pressure; ecg; GCS; operative severity; multiple procedures, total blood loss, peritoneal soiling, finding of peroperative malignancy; elective or acute surgery, WBC, Hb, urea, potassium, sodium

Peptic ulcer perforation score (PULP score).

American society of anesthesiologists (ASA) score.

Acute physiology and chronic health evaluation II (APACHE II).

Simplified acute physiology score II (SAPS II).

Mortality probability models II (MPM II).

Physiological and operative severity score for the enumeration of mortality and morbidity (POSSUM) score.

Glasgow coma scale (GCS).

White blood cell count (WBC).

Gastrointestinal bleeding (GI bleeding).

Hemoglobin (Hb).

### Scoring systems aimed at prediction of outcome in PPU

The Boey score was the first score directly aimed at mortality prediction for perforated peptic ulcer [14]. The original work by Boey et al stated that delay of surgery after onset of symptoms for more than 48 hours, shock upon admission and a high degree of comorbidity, were associated with a 100% mortality when all factors where present. Eventually, the delay of surgery was adjusted to 24 hours, and the scoring system was validated in a cohort from Hong Kong [14,28].

The Hacettepe score was also developed for PPU patients and comprises four factors (Table 1) [17]. This study evaluated 173 patients from Turkey and found the Hacettepe score to be equivalent to the Mannheim Peritonitis Index (MPI), with a sensitivity of 83% and specificity of 94% for mortality prediction. The sensitivity for the MPI in this study was 75% and the specificity 96%. Eventually, this score was used in a study from India, as elaborated below [17,18].

The Jabalpur score was based on a study on 140 patients from India, with a mean age of 39 years. This score takes into account six factors, which are all assessable preoperatively. Both morbidity and mortality were predicted accurately, based on a high AUC value (Table 2) [18].

**Table 2:** Scoring accuracy of mortality prediction in PPU patients

**Scoring systems evaluated**	**Mishra*et al***[18]	**Lohsiriwat *et al ***[10]	**Koc *et al ***[16]	**Møller *et al ***[19]	**Buck *et al ***[22]
	**Mortality rate**	**Mortality rate**	**Mortality rate**	**Mortality rate**	**Mortality rate**
	**10.7%**	**9.0%**	**10.6%**	**27.0%**	**17.0%**
	**Area under the ROC Curve (AUC)**				
ASA		0.91		0.78	0.73
Boey	0.85	0.86		0.70	0.63
Apache II			0.87		0.76
SAPS II			0.86		
MPM II			0.98		
Hacateppe score	0.72				
Jabalpur score	0.92				
MPI		0.84			
Modified Apache II	0.84				
Modified MPI	0.85				
PULP				0.83	
Sepsis score					0.69

AUC denotes area under the curve . An AUC value > 0.8 is considered good, a range between 0.60-0.80 is considered as moderate, and an AUC value < 0.60 is regarded as poor.

*ASA*, American society of anesthesiologists (ASA) score.

APACHE II denotes Acute physiology and chronic health evaluation II.

Modified *APACHE II*, APACHE II calculated when no available blood gas.

S*APS*, Simplified acute physiology score II (SAPS II).

*MPM*, Mortality probability models II (MPM II).

MPI denotes Mannheim peritonitis index.

*Modified MPI*, MPI calculated when no available blood gas.

Most recently, the Peptic Ulcer Perforation (PULP) score has been introduced as a scoring system for perforated peptic ulcer. This score is based on a nationwide study from Denmark and included 2668 PPU patients with a median age of 70.9 years, where 55% was female. Seven factors are taken into account, with weighted points applicable for each factor, with a maximum sum of 18 points being the highest possible. The optimal cut-off point was found to be 7 points, which gives a positive predictive value (PPV) of 25% for those with 0-7 points, and a PPV of 38% for the group with 8 or more points [19]. The PULP study also compared different systems elaborated below.

### General scores of comorbidity

The ASA score introduced in 1941 and intended for preoperative assessment of patients’ fitness level, is the oldest available scoring system [20,29]. ASA score is frequently reported together with other descriptive patient data including age, gender, and various physiologic parameters, but this classification has no specific role in outcome prediction of patients with PPU per se.

The Charlson comorbidity index was developed to stratify comorbidity into different risk groups by assigning scores to various illnesses [21]. The Charlson index is a widely used scoring system and considers 19 conditions deemed clinically important, and they are each given 1 to 6 points due to high or low morbidity grade. Cerebrovascular disease is given 1 point, severe liver disease 3 points and metastatic cancer and AIDS are given 6 points. The Charlson index was initially suggested for prediction of long-term mortality. However, later studies have found it to be useful also in prediction of in-hospital morbidity and mortality [30,31]. One study also used the Charlson comorbidity index to predict outcome in PPU patients. A highly significant association between a medium or high Charlson score and 30-day mortality was observed, with an odds ratio (OR) of 4.17 for high score (3 or more points on the Charlson score) and an OR of 3.99 for medium score (1-2 points on the Charlson score) [32]. However, identification of any other PPU studies to confirm the obtained results by use of this particular score was not possible.

The Sepsis criteria are easy to calculate preoperatively and the presence of sepsis is fulfilled if two or more of the following parameters are present, when infection is confirmed or highly likely; temperature >38°C or <36°C respiratory rate > 20 per minute or PCO_2_ < 4.3kPa, heart rate > 90 per minute, White cell count > 12.0×109 or < 4.0×109 [33].

Obviously this system is widely used in several aspects of medicine, but has also been applied to predict outcome in a PPU cohort, as elaborated below [22].

The Mannheim Peritonitis Index (MPI) consists of seven factors that are more directly related to the operative findings. As the name implies, the design was specifically intended for surgical patients presenting with peritonitis. It comprises both preoperative and perioperative conditions, and has been found to predict morbidity well, but less so in prediction of mortality for PPU patients [10,23].

### Intensive care unit (ICU) systems

The Acute physiology and chronic health evaluation II (APACHE II) score is a common score globally and the most used ICU scoring system in the USA. It comprises twelve different physiological measurements, age and previous health status, and was originally designed to categorize ICU patients according to risk. The system gives an increasing amount of points for extreme values (high or low), between 0 (36.0°C -38.4°C) and 4 (≥41°C and ≤29.9°C). Originally this score was found to perform well amongst ICU patients [24]. Later, it was also applied to predict outcome in PPU patients. One study from the USA [34] reported on zero mortality in PPU patients with scores less than 11 points, and a 35% mortality rate in patients with at least 11 points, which indicates this as a useful cut-off. Others have tried different cut-off values without finding these to be more useful. However the APACHE II score is a rather complex system needing mathematical equations to calculate and a minimum of 24 hours to assess all factors. This may pose implications and concerns for its clinical usefulness and availability. Nevertheless, APACHE II has been shown to predict outcome well also for PPU patients [16,24,34].

The Simplified acute physiology score II (SAPS II) is designed for predicting outcome in ICU patients and consists of 17 variables. It was developed in the 80s and a revised version was introduced in 1993 [25]. The SAPS II system is frequently used for outcome prediction in critically ill patients in Europe and Scandinavia, and has many similarities with the APACHE II system [25]. Both systems are rather complex, with a number of factors incorporated in the calculations, including physiologic parameters. The SAPS II system predicts mortality and morbidity well, but also seems more suitable for ICU patients. Nevertheless, this score performed well for outcome prediction of PPU patients [16].

The Mortality probability models II (MPM II) was designed for prediction of outcome in ICU patients. MPM II assesses the presence or not of 14 different clinical factors, several related to systemic perfusion [26]. The MPM II predicted mortality better then both SAPS II and APACHE II in one study [16]. However this study was rather small and skewed with basically younger male patients, which is in contrast to current PPU cohorts. While some studies from Asia and Africa have presented similar patient characteristics [35,36], recent studies from Scandinavia and Northern Europe have presented data with a 1:1 male/female ratio and median age close to 70 years [19,27]. Moreover, the MPM II is a rather complex system, thus suboptimal for a pre-operative calculation in the clinical context PPU patients present.

The Physiological and Operative Severity Score for Enumeration of Mortality and Morbidity (POSSUM score) consists of 12 factors based on the patients physiological state and 6 factors regarding operative conditions. These factors are then entered into two mathematical equations for risk assessment [37]. The POSSUM score was designed for outcome prediction in ICU patients, and is widely used in the UK. The original POSSUM score tended to overestimate mortality for low risk groups. The Portsmouth-POSSUM (P-POSSUM) was therefore designed to adjust for this [38]. Prediction of mortality in patients undergoing emergency laparotomy was improved by the P-POSSUM score, compared to the original POSSUM score [38,39]. The POSSUM-phys score, which is the physiologic subscore, comprises only the 12 physiologic parameters, which can be assessed preoperatively. Only one study was found that applied POSSUM-phys to PPU patients. In this study 261 PPU patients with a mean age of 67 years were evaluated and the POSSUM-phys score predicted both mortality and morbidity [27]. The POSSUM-phys score, in contrast to the POSSUM score, can be assessed pre-operatively. However, with regard to PPU patients, we have not encountered any studies comparing POSSUM-phys score with other scoring systems.

### Isolated risk factors for morbidity and mortality in PPU

In a large systematic review of pre-operative prognostic factors in PPU patients, Møller et al identified 50 prognostic studies evaluating overall 37 prognostic factors in a population total of 29,782 patients [40]. They deemed the overall methodological quality to be acceptable, yet only two-thirds of the studies provided confounder-adjusted estimates in the multivariable analyses. Very few of the included studies investigated all, or the majority of, the prognostic factors included in the review. Some of the markers were only investigated in a few studies, and overall, the diversity and spread across the studies were considerable for most markers.

Nonetheless, adjusted pooled relative risks showed evidence for an association between mortality and older age, comorbidity, and the use of medications such as Non Steroid Anti Inflammatory Drugs (NSAIDs), steroids and immunosuppressives [40]. Further predictive factors associated with a poor prognosis included shock upon admission, pre-operative metabolic acidosis, tachycardia, elevated respiratory rate, acute renal failure, low serum albumin level, high ASA score, and a pre-operative time-delay >24 hours. Notably, several of the scoring systems proposed (Table 1) include one or several of these factors, but usually not all.

### Studies comparing several scoring systems for outcome prediction in PPU patients

Five studies were identified where AUC values were compared between different scoring systems. Mortality prediction varied from 0.63 to 0.98 for the different systems evaluated in these studies and these are presented in Table 2[10,16,18,19,22].

In another study from Chicago, including 436 PPU patients, the Boey score was compared to the APACHE II score [41]. The Boey score predicted mortality, but failed to predict morbidity. Moreover, the Boey score predicted conversion from laparoscopy to laparotomy, with a conversion encountered in 81.8% of Boey 2 score patients. The APACHE II was found to predict both morbidity and mortality. However, no AUC or relative risks were calculated and of such it is not directly comparable to other studies [41].

A report from Finland in the early 2000s included 280 PPU patients [23] and MPI predicted postoperative morbidity better than both the ASA and the Boey score. But the Boey score predicted mortality better than both the ASA score and the MPI. The authors used likelihood ratios to discriminate, but no AUC testing was done in this study.

### Accuracy of morbidity prediction

We identified only two studies that reported AUC values for morbidity prediction [10,22]. Lohsiriwat et al defined morbidity as some form of complication and found AUC values of 0.80 for both the ASA and the Boey score, while MPI performed poorest with an AUC of 0.74 [10]. Buck et al defined septic shock and ICU admission as secondary endpoints and found following AUC values; for septic shock the AUC values were 0.67 for the ASA score, 0.72 for the Boey score, 0.74 for the sepsis score and 0.78 for the APACHE II score. For ICU admission the AUC values were 0.69 for the ASA score, 0.64 for the Boey score, 0.72 for APACHE II score and 0.64 for the sepsis score. Overall the APACHE II performed best for prediction of the two chosen secondary endpoints.

An overview of scoring systems, with mortality figures, published in various studies since the year 2000, are presented in Table 3[4,7,10,16,18,19,23,27], [32,35,36,42-59].

**Table 3:** PPU studies reporting on 30-day mortality

**Study (reference)**	**Country**	**Study period**	**Number of patients**	**Mortality rate, n (%)**	**Age, mean**	**Score system used**
Arici et al [50]	Italy	1991-2004	147	14%	52 (median)	Boey
Arveen et al [36]	India	2006-2008	328	9%	43	ASA
Bae et al [7]	Korea	2006-2007	4258	3%	74% younger than 60 years of age	No score system
Bas et al [51]	Belgium	1998-2004	97	5%	39	No score system
Bin-Taleb et al [52]	Yemen	1997-2006	156	4%	39	No score system
Chalya et al [56]	Tanzania	2006-2011	84	11%	28 (median)	No score system
Christensen et al [2]	Denmark	1991-2003	2061	25%	61% older than 65 years of age	No score system
Dakubo et al [35]	Ghana	1998-2002	326	11%	41	No score system
Egberts et al [27]	Germany	1993-2005	261	24%	67 (median)	Possum-phys
Forsmo et al [46]	Norway	1992-2003	102	22%	71 (median)	ASA
Hemmer et al [43]	The Netherlands	2000-2005	272	16%	62	ASA
Kamani et al [57]	Iran	1996-2005	56	5%	50	No score system
Kim et al [49]	Korea	2005-2010	142	6%	57	ASA
Koc et al [16]	Turkey	2005-2006	75	11%	44 (median)	APACHE II, APACHE III SAPS II, MPM II
Kocer et al [47]	Turkey	2001-2004	269	9%	43	ASA
Kujath [48]	Germany	1996-2000	102	14%	69	ASA
Larkin et al [45]	Ireland	1998-2007	76	20%	60	ASA
Lohsiriwat et al [10]	Thailand	2001-2006	152	9%	52	Boey, ASA, MPI
Makele et al [23]	Finland	1979-2000	280	14%	58	Boey, ASA, MPI
Mishra et al [18]	India	1999-2001	140	11%	39	Apache II
						MPI
						Jabalpur
Montalvo-Jave et al [53]	Mexico	2006-2008	30	17%	57	No score system
Muslu et al [58]	Turkey	1998-2005	126	4%	51	No score system
Møller et al [19]	Denmark	2003-2009	2668	27%	71 (median)	PULP, ASA, Boey
Nasio/Saidi [59]	Kenya	2005-2006	44	9%	35	No score system
Noguiera et al [44]	Portugal	1990-2000	210	10%	53 (median)	No score system
Rajesh et al [55]	India	2006-2011	180	13%	<50 years	Apache II
						Boey
Subedi et al [54]	Nepal	2002-2004	145	7%	46	No score system
Taha et al [32]	Scotland	1997-2006	270	19%	64 (median)	Charlson comorbidity index
Thorsen et al [4]	Norway	2003-2009	114	16%	67 (median)	ASA
						Boey

Table sorted alphabetically by study author’s name.

Mortality rates and age are rounded up/down.

American society of anesthesiologists (ASA) score.

Acute physiology and chronic health evaluation II (APACHE II).

Simplified acute physiology score II (SAPS II).

Mortality probability models II (MPM II).

Mannheim peritonitis index (MPI).

Peptic ulcer perforation (PULP) score.

Physiological and operative severity score for the enumeration of mortality and morbidity physical sub-score (POSSUM-phys score).

## Discussion

In this review we identified four scoring systems that have been developed specifically for prediction of outcome in PPU patients. However, several other general scores have been applied on this particular group of patients, although these scores have a non-specific design for PPU patients. We also identified several factors that make direct comparison of results and pooling of patient populations for assessment of outcome prediction difficult or impossible. Among these obstacles are time and sociodemographic differences and differences in score design and complexity. Some factors of these barriers are further discussed below.

### Implications of age on score performance

The Boey score was developed from a study population with a median age of 51 years [14]. However, the age of PPU patients in recent studies from Europe have been considerably higher [19,27,43,45], and age has been shown to be an isolated predictor for mortality in PPU patients [4,19,40,43]. Also, in the identified studies on mortality of PPU patients published the last decade, the 30 day mortality was at least 14% when age was mean or median > 60 years [4,19,27,32,43,45,48], in contrast to a decreased mortality between 3% and 14% in patient series with a mean or median age of < 60 years [7,10,16,23,36,44,47]. Hence, the Boey risk score may not be as suitable for the older age groups as for the younger. As pointed out by others [22], there is also a weakness in its crudeness, including the definition of shock with a systolic blood pressure < 90 mm Hg in the original study by Boey. In contrast, shock is usually defined by a combination of systolic blood pressure ≤100 mm Hg and tachycardia, defined as a pulse ≥100 per minute [19,43]. Thus, the Boey score may vary due to the definitions used. Nevertheless, several studies have found the Boey score to be a good predictor of mortality in PPU patients [10,19,23]. The Boey score was specifically designed for PPU patients, and while definitions are of some concern, the simplicity makes it very quick to calculate which is an advantage. Of note, the Boey score has not performed as well in predicting morbidity [10,60,61].

### Differences in sociodemographic regions

The Hacettepe score has been applied in two different studies with varying results and not better than the other systems used. It evaluates four factors and should be feasible to assess. However, 77% of the patients were < 50 years of age and 94% were men. Similar PPU patient groups are found in other developing countries, but the patient demography is quite different in non-developing countries, with older age and minor differences between genders.

The Jabalpur score performed a high AUC score in the only study reporting it [18]. But the patient group was among the youngest with a mean age of 39 years with 98% males. Therefore, it seems more applicable in regions with similar demography. When these two systems were tested in cohorts in India and Turkey, the Jabalpur system performed superior to the Hacettepe score [17,18].

The nationwide PULP study is the largest recent study evaluating outcome prediction for PPU patients. Hence the external validity may be stronger, at least for comparable, western populations with demography similar to Denmark. The PULP score incorporates both the ASA score and the Boey score and can be evaluated pre-operatively. Since it does incorporate both these systems, one would expect the PULP score to perform at least as well as the other systems on their own. Indeed, the PULP score did perform somewhat better, with slightly higher AUC values than both the ASA score and the Boey score in predicting morbidity and mortality [19]. However a validation of this system is needed.

Furthermore, the PPU systems found in the literature and presented in this review differ in many ways. The Boey score [14]was originally done on American patients in the early 80s, the Hacettepe score [17] on a Turkish population in the early 90s, the Jabalpur score [18] on an Indian population in the early 2000s and the PULP score [19] in Denmark in the late 2000. These studies differ in geography, mean age and decade presented, which means that further validation is recommended before any scoring system can be applied to a cohort different from the population of development.

### Comparing AUC values

When comparing AUC values from ROC analyses from different studies, several limitations have to be considered. For one, different inclusion criteria and patient characteristics will potentially bias direct comparison of AUC values among studies. Hence, comparing ROC curve analysis and AUC values is best done on the same mix of patients [62]. A prediction model is made from fitting the data in a patient series by the means of logistic regression. Data included in such models may differ between studies, which may hamper comparison of AUC values of scoring systems from different studies.

Second, the number and the ratio of outcome events to the study population will influence the AUC accuracy and its precision. The PULP study included 2668 patients and with a mortality rate of 27% (n = 720 patients) as the evaluated outcome. Too few such outcome events in any given study will make logistic regression problematic and unreliable, with the confounding factors having a greater influence and thus a bias on the results. As a comparably large sample size, the PULP study may have higher power and reliability, than the other four studies comparing AUC values. However, the patient characteristics in the PULP cohort differ from the other studies, which may influence the external validity. Thus, this model may not necessarily work well for other cohorts, and should thus be tested externally.

Lastly, timing of collection of the score variables is another important issue to consider when a scoring system for outcome prediction should be chosen. The more information that is available, the better would the performance of the score be expected. Hence a post-operative score would likely perform better as compared to a pre-operative or a peri-operative scoring system.

### Complexity of scores

The ASA score is in contrast to the ICU systems simple to calculate, and it has been shown in various studies to predict both morbidity and mortality in general surgical patients [10-13,19]. Even though the ASA score neither was designed for, nor solely functions as a PPU score, it is widely used in PPU studies evaluating outcomes [4,10,19]. The ASA score has been shown to predict mortality well in several groups of patients, included for PPU patients [10,18,19,47]. The AUC values reported vary from 0.73 to 0.91, but the vast majority does not report AUC values or other parameters that make it possible to compare different studies. The main problem of the ASA score has been the inter-observer variability [63,64].

The MPI is a more complex system and must be obtained during or after surgery, which is regarded challenging. However it has been shown to predict both morbidity and mortality, although to a varying degree. Only one study reported an AUC value for MPI (0.84), which is considered good [16], but both the ASA score and the Boey score performed better in this study [10].

Four ICU systems that were developed for outcome prediction of critically ill patients have been applied to PPU patients in one or more studies. In addition one study used the Charlson comorbidity index. But the complexity of these systems seems to limit the implementation in a general clinical setting. The APACHE II score is most frequently reported, but is still no common system used for PPU patients in general. They have all been shown to predict outcome for PPU patients, but to varying degree. And since only the fewest studies actually report on AUC values or other values that can be compared to others, data are sparse. MPM II performed the highest AUC value of all the scoring systems regarding mortality prediction with 0.98, which is nearly perfect. However, this study was small and skewed, with mostly younger male patients, with a low mortality rate. Hence, its validity to patients with a different age and demographic patterns is highly questionable.

The Sepsis score has an important status in emergency medicine and is easy and rapid to calculate. Early recognition of the systemic inflammatory response syndrome (SIRS) and prompt goal directed therapy, including perioperative and postoperative, can be of paramount importance and may influence outcome [9,33]. Probably, treatment delay in PPU patients, one of the factors in both the Boey score and the PULP score, is actually a surrogate marker for imminent sepsis. Also the presence of electrolyte disturbances, hypoalbuminemia, anemia, kidney failure, leukocytosis and shock can all be seen as part of the sepsis syndrome in a condition like perforated peptic ulcer. The sepsis score has also been found to predict outcome in PPU patients, but less so than most of the other scores evaluated [22].

## Conclusions

While no scoring system was ideal and all were hampered by certain limitations, a few scores appeared easily applicable in clinical practice. The Boey score and the ASA score are most commonly applied in the current literature to predict outcomes for PPU patients, but both demonstrate variable accuracy. While the PULP score seems promising, a validation is pending before a general application can be recommended.

## Abbreviations

PPU: Perforated peptic ulcer; ASA score: American society of anesthesiologists score; ROC: Receiver operating characteristics curve; AUC: Area under the curve; MPI: Mannheim peritonitis index; APACHE II: Acute physiology and chronic health evaluation II; SAPS II: Simplified acute physiology score II; MPM II: Mortality probability models II; POSSUM- phys score: Physiological and operative severity score for the enumeration of mortality and morbidity phys score; OR: Odds ratio; ICU: Intensive care unit; NSAIDS: Non steroid anti inflammatory drugs; SIRS: Systemic inflammatory response syndrome.

## Competing interests

The authors declared that they have no competing interest.

## Authors’ contributions

KT and KS planned and designed the study. KT did the litterature search and drafted the manuscript. KS and JAS revised the article. All authors read, revised and approved the final manuscript.
